# FDG PET, dopamine transporter SPECT, and olfaction: Combining biomarkers in REM sleep behavior disorder

**DOI:** 10.1002/mds.27094

**Published:** 2017-07-22

**Authors:** Sanne K. Meles, David Vadasz, Remco J. Renken, Elisabeth Sittig‐Wiegand, Geert Mayer, Candan Depboylu, Kathrin Reetz, Sebastiaan Overeem, Angelique Pijpers, Fransje E. Reesink, Teus van Laar, Lisette Heinen, Laura K. Teune, Helmut Höffken, Marcus Luster, Karl Kesper, Sofie M. Adriaanse, Jan Booij, Klaus L. Leenders, Wolfgang H. Oertel

**Affiliations:** ^1^ Department of Neurology University of Groningen, University Medical Center Groningen Groningen The Netherlands; ^2^ Department of Neurology Philipps‐Universität Marburg Marburg Germany; ^3^ Neuroimaging Center Department of Neuroscience, University of Groningen Groningen The Netherlands; ^4^ Department of Neurology and JARA‐BRAIN Institute Molecular Neuroscience and Neuroimaging Aachen University Aachen Germany; ^5^ Kempenhaeghe Foundation, Sleep Medicine Centre Heeze The Netherlands; ^6^ Department of Nuclear Medicine Philipps‐Universität Marburg Marburg Germany; ^7^ Department of Internal Medicine Section Respiratory Diseases, Philipps Universität Marburg Marburg Germany; ^8^ Department of Nuclear Medicine Academic Medical Center Amsterdam The Netherlands; ^9^ Hephata Klinik Schwalmstadt Germany; ^10^ Institute for Neurogenomics, Helmholtz Center for Health and Environment München Germany

**Keywords:** idiopathic REM sleep behavior disorder, Parkinson's disease‐related pattern, ^18^F‐FDG‐PET, dopamine transporter ^123^I‐FP‐CIT SPECT, olfaction

## Abstract

**Background:**

Idiopathic REM sleep behavior disorder is a prodromal stage of Parkinson's disease and dementia with Lewy bodies. Hyposmia, reduced dopamine transporter binding, and expression of the brain metabolic PD‐related pattern were each associated with increased risk of conversion to PD. The objective of this study was to study the relationship between the PD‐related pattern, dopamine transporter binding, and olfaction in idiopathic REM sleep behavior disorder.

**Methods:**

In this cross‐sectional study, 21 idiopathic REM sleep behavior disorder subjects underwent ^18^F‐fluorodeoxyglucose PET, dopamine transporter imaging, and olfactory testing. For reference, we included ^18^F‐fluorodeoxyglucose PET data of 19 controls, 20 PD patients, and 22 patients with dementia with Lewy bodies. PD‐related pattern expression *z*‐scores were computed from all PET scans.

**Results:**

PD‐related pattern expression was higher in idiopathic REM sleep behavior disorder subjects compared with controls (*P* = 0.048), but lower compared with PD (*P* = 0.001) and dementia with Lewy bodies (*P* < 0.0001). PD‐related pattern expression was higher in idiopathic REM sleep behavior disorder subjects with hyposmia and in subjects with an abnormal dopamine transporter scan (*P* < 0.05, uncorrected).

**Conclusion:**

PD‐related pattern expression, dopamine transporter binding, and olfaction may provide complementary information for predicting phenoconversion. © 2017 The Authors. Movement Disorders published by Wiley Periodicals, Inc. on behalf of International Parkinson and Movement Disorder Society.

Longitudinal studies have shown that >80% of individuals with idiopathic REM sleep behavior disorder (RBD) developed Parkinson's disease (PD) or dementia with Lewy bodies (DLB) on long‐term follow‐up.^1^, ^2^, ^3^, ^4^, ^5^ RBD subjects represent a suitable group to study the prodromal stage of these disorders and may be crucial for disease‐modification trials. However, such trials require biomarkers that can reliably identify at‐risk individuals and predict clinical manifestation of PD/DLB.

Neurodegenerative disorders are characterized by disease‐specific patterns of altered brain glucose metabolism on ^18^F‐fluorodeoxyglucose positron emission tomography (^18^F‐FDG‐PET) brain imaging. Such patterns can be extracted from ^18^F‐FDG‐PET data with scaled subprofile model and principal component analysis (SSM PCA^6^). With SSM PCA, a PD‐related pattern (PDRP) has been identified in multiple cohorts.^7^, ^8^, ^9^, ^10^ The degree to which the PDRP is present in a new ^18^F‐FDG‐PET scan can be quantified, resulting in a subject score. PDRP subject scores increase with disease progression and decrease with effective therapy.^10^, ^11^

To date, 2 groups have reported that RBD subjects have higher PDRP subject scores compared with controls.^12^, ^13^ In a longitudinal study of 17 RBD subjects, baseline PDRP expression was associated with a high risk of developing PD or DLB within 5 years.^12^

Other markers have also been considered. Loss of striatal dopamine transporter (DAT) binding on single photon emission computed tomography (DAT‐SPECT) indicates imminent phenoconversion.^14^, ^15^ In addition, RBD subjects with baseline hyposmia have a high risk of developing PD/DLB within 5 years of follow‐up.^16^, ^17^

The PDRP has potential as a disease biomarker in prodromal subjects, but further validation by an independent research group is essential. Moreover, direct comparisons between PDRP expression, DAT binding, and olfaction in the same RBD subjects have never been made. We therefore studied these 3 markers in 21 RBD patients.

## Methods

Twenty‐one subjects with RBD (polysomnographically confirmed^18^) were evaluated with ^18^F‐FDG‐PET, DAT‐SPECT, and olfactory testing. Per inclusion criteria, RBD subjects did not have parkinsonism^19^ or DLB^20^ at the time of the study. Participants with a history of psychotropic medication use before the onset of RBD were excluded.^21^

Nineteen age‐matched healthy controls were studied with ^18^F‐FDG‐PET and olfactory testing. Controls did not have RBD (score < 5 on the RBD screening questionnaire^22^) and furthermore had no first‐degree family members with a neurodegenerative disease.

RBD subjects and controls were investigated with the Unified Parkinson's Disease Rating Scale (UPDRS, version 2003^23^) and the Montreal Cognitive Assessment (MoCA).^24^ Olfactory function was assessed with Sniffin' Sticks.^15^, ^16^, ^25^ Total olfaction scores (TDI) were obtained by summing the threshold (T), discrimination (D), and identification (I) subscores. Five olfactory stages were defined as follows: anosmia, TDI ≤ 15; severe hyposmia, 15 < TDI ≤ 20); moderate hyposmia, 20 < TDI ≤ 25; mild hyposmia, 25 < TDI ≤ 30; and normosmia, TDI > 30. In a previous study, it was determined that a baseline TDI score < 18 was associated with increased risk of phenoconversion to PD/DLB within 5 years of follow‐up.^16^ We therefore divided RBD patients into 2 groups: patients with TDI scores < 18 and patients with TDI scores ≥ 18.

For reference, we studied the ^18^F‐FDG‐PET scans of retrospectively‐included patients with clinical diagnoses of “probable PD” (n = 20, nondemented, aged 67.5 ± 8.6 years; 16 men; median disease duration, 2 years; interquartile range, 1‐7 years) and “probable DLB” (n = 22, aged 73.7 ± 7 years; 17 men; median disease duration, 3 years; interquartile range, 1‐4 years) according to consensus criteria.^19^, ^20^

Exclusion criteria for all subjects included a history of (other) neurological diseases, diabetes mellitus, stroke, significant head trauma, or other relevant comorbidities. The study was approved by local institutional review boards. Voluntary written informed consent was obtained from each subject after verbal and written explanation of the study, in accordance with the Declaration of Helsinki.

All subjects underwent static ^18^F‐FDG‐PET imaging on a Siemens Biograph mCT‐64 PET/CT camera (Siemens, Munich, Germany) at the University Medical Center Groningen, the Netherlands. Images were reconstructed with OSEM3D, including point‐spread function and time‐of‐flight modeling, and smoothed with a Gaussian 8‐mm full‐width at half‐maximum filter. Central nervous system depressants were discontinued in all subjects for at least 24 hours before each scan. In RBD patients, all RBD‐related medications (eg, melatonin or clonazepam) were discontinued for at least 48 hours prescan. In PD and DLB patients, dopamimetics were not withheld.

All images were spatially normalized onto an ^18^F‐FDG‐PET template in Montreal Neurological Institute brain space^26^ using SPM12 software (Wellcome Department of Imaging Neuroscience, Institute of Neurology, London, UK) implemented in Matlab (version 2012b; MathWorks, Natick, Massachusetts). Expression of the previously identified PDRP^8^ was calculated in the new ^18^F‐FDG‐PET data as described previously.^27^ All PDRP subject scores were *z*‐transformed to the controls (n = 19), such that the average PDRP *z*‐score in controls was 0, with a standard deviation of 1.

In future clinical trials of RBD, diagnostic tool specificity will be more important than sensitivity (ie, RBD subjects who will not phenoconvert should be excluded). We therefore reanalyzed the PDRP identification cohort^8^ and selected a cutoff *z*‐score that gave 100% specificity. At PDRP *z* = 1.8, there was no misclassification of controls in the identification cohort (data not shown). This threshold was applied to the PDRP *z* scores in the current study (ie, a *z* score ≥ 1.8 was considered indicative of PD).

RBD subjects underwent DAT imaging with ¹²³I‐2β‐carbomethoxy‐3β‐(4‐iodophenyl)‐N‐(3‐fluoropropyl)nortropane single photon emission computed tomography. DAT binding in striatal regions was quantified with the Brain Registration & Analysis Software Suite (BRASS; HERMES Medical, Sweden). Non‐specific‐binding ratios were calculated in the caudate nucleus and putamen bilaterally, using the occipital cortex for reference (ie, nonspecific binding). DAT‐binding ratios that were 2 or more standard deviations lower than age‐matched expected control values were considered abnormal (see Supplementary Material). The lowest putamen DAT‐binding ratio of each subject was used for further analyses. The median interval between acquisition of the ^123^I‐FP‐CIT‐SPECT and ^18^F‐FDG‐PET was 2.7 months (interquartile range, 1.3‐4.6 months; total range, 12 days to 9.6 months). Loss of striatal DAT binding in the putamen was considered abnormal for age in 9 of 21 RBD subjects.

### Statistical Analysis

The normality of distribution of each variable was assessed with the Shapiro‐Wilk test, Q‐Q plots, and box plots. PDRP *z*‐scores and DAT‐binding ratios were parametric. PDRP *z*‐scores were compared across controls, RBD, PD, and DLB with a 1‐way analysis of variance (ANOVA) with post hoc Bonferroni corrections.

PDRP *z*‐scores were compared between RBD subjects with normal and abnormal DAT scans with an independent *t* test. PDRP *z*‐scores and DAT‐binding ratios were also compared between the 2 olfaction categories (TDI score < 18 or ≥ 18) with an independent *t* test. These analyses were not corrected for multiple comparisons.

In the 21 RBD subjects, correlations between PDRP *z*‐scores and DAT‐binding ratios were tested for significance with a Pearson correlation coefficient. TDI, MoCA, and UPDRS‐III scores were nonparametric. Correlations between these variables and the imaging metrics (PDRP *z*‐scores and DAT binding) were assessed with a Spearman's rank correlation coefficient. Correlations were considered significant at *P* < 0.05 (uncorrected). All analyses were performed using SPSS software version 23 (SPSS Inc., Chicago, IL).

## Results

UPDRS‐III scores were significantly higher in RBD subjects compared with controls. MoCA and olfaction scores were significantly lower in RBD patients (*P* < 0.01; Supplementary Table).

**Figure 1:**
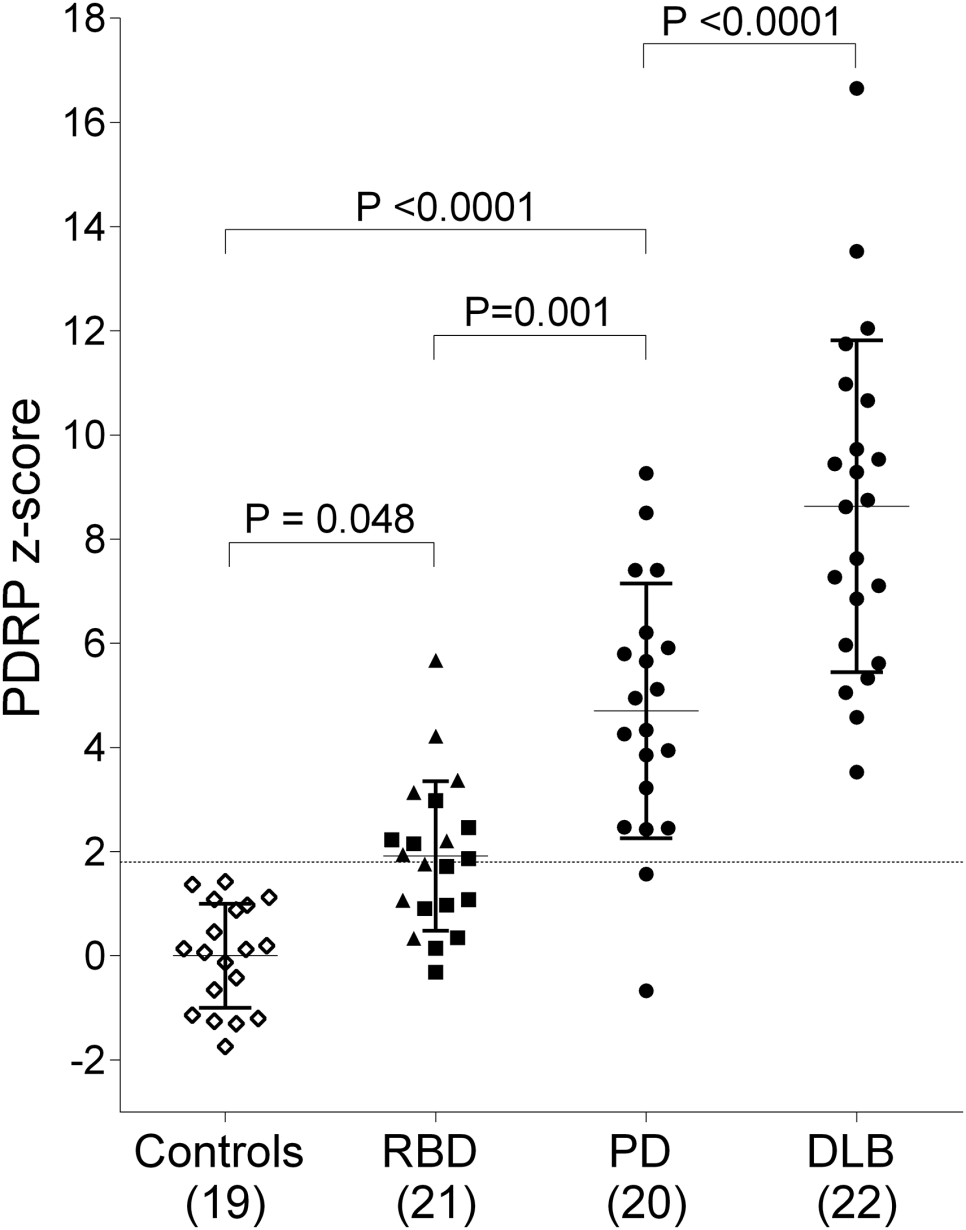
PDRP *z* scores across groups. PDRP expression was calculated in all groups and *z*‐transformed to the healthy controls. PDRP expression *z* scores were compared across groups with a 1‐way analysis of variance. Post hoc comparisons were Bonferroni‐corrected. The dashed line (*z* = 1.8) indicates the cutoff for PDRP expression. Triangles indicate RBD subjects with abnormal DAT scans. Squares indicate subjects with normal DAT scans.

PDRP subject scores were not significantly different between men (n = 9) and women (n = 10) in the control group (*P* = 0.75, independent *t* test). Stepwise increases in PDRP *z* scores were observed across groups (ANOVA *F*_81_ = 59.06, *P* < 0.0001; Fig. 1). In 12 of 21 RBD subjects (57%), the PDRP *z* score surpassed the threshold (*z* ≥ 1.8; Table 1).

**Table 1:** Clinical and imaging characteristics of the 21 RBD subjects

PDRP *z* score category	DAT scan category	RBD subject	PDRP *z* score	Lowest putamen DAT‐binding ratio	Total olfaction score (TDI)^b^	Sex	Age	RBD duration (years)	Age at onset RBD	MoCA	UPDRS‐III
<1.8	Normal	1	1.7	2.0^c^	33.8	Male	57.4	5.0	52.4	30.0	4.0
		2	1.0	2.3	33.5	Female	58.9	7.0	51.9	27.0	0.0
		3	‐0.3	2.5	33.5	Female	68.3	6.0	62.3	23.0	2.0
		4	1.1	2.5	29.5	Male	54.0	6.0	48.0	26.0	4.0
		5	0.9	2.4	28.0	Male	56.4	6.0	50.4	27.0	1.0
		6	0.2	2.2	19.5	Male	67.1	25.0	42.1	28.0	0.0
		7	0.4	2.9	0.0	Male	56.0	9.0	47.0	25.0	1.0
	Abnormal	8	0.3	1.2	19.0	Male	65.9	12.0	53.9	26.0	2.0
		9	1.1	1.0	13.0	Male	66.4	6.0	60.4	27.0	3.0
≥1.8	Normal	10	2.2	2.5	29.0	Male	57.8	5.0	52.8	28.0	1.0
		11^a^	2.2	2.3	23.5	Male	62.6	14.0	48.6	24.0	5.0
		12	3.0	2.5	20.5	Male	57.5	2.5	55.0	27.0	6.0
		13	1.9	2.3	16.5	Male	64.5	2.0	62.5	26.0	2.0
		14^a^	2.5	2.0^c^	15.5	Female	70.1	3.0	67.1	28.0	4.0
	Abnormal	15	2.2	1.7	27.5	Male	64.0	14.0	50.0	28.0	4.0
		16	1.8	1.6	25.8	Male	66.9	3.0	63.9	27.0	2.0
		17	3.4	0.9	17.0	Male	61.5	4.0	57.5	27.0	0.0
		18	3.1	1.7	13.0	Male	65.4	6.0	59.4	27.0	6.0
		19	4.2	2.0^c^	2.0	Male	49.9	4.0	45.9	24.0	1.0
		20	5.7	1.2	0.0	Male	63.2	4.0	59.2	28.0	1.0
		21	1.9	1.8	0.0	Male	66.6	2.0	64.6	22.0	5.0

a In these 2 RBD subjects, ^18^F‐FDG‐PET was performed respectively 3.4 and 1.5 months before DAT‐SPECT.

^b^Olfaction was measured with the Sniffin' Sticks test; total TDI scores are reported in this table (see main text). A TDI > 30 indicates normal olfactory function; a TDI ≤ 20 indicates severe hyposmia. A TDI score < 18 was previously associated with an increased risk of phenoconversion to PD/DLB.^16^

^c^Subjects 1, 14, and 19 all have putamen DAT‐binding ratios of 2.0. Subjects 1 and 14 are still in the “normal DAT” category, and subject 19 is in the “abnormal DAT” category. This is because DAT‐binding ratios were considered abnormal if they were 2 standard deviations below the value expected for age. For subjects 1 and 14, the ratio of 2.0 is still normal for age (57 and 70 years old, respectively); however, for subject 19, this ratio is abnormal for age (50 years old). We note that subject 1 has a borderline‐normal DAT‐binding ratio and PDRP *z*‐score (*z* = 1.7).

RBD, idiopathic REM sleep behavior disorder; PDRP, Parkinson's disease‐related pattern; DAT, dopamine transporter; MoCA, Montreal Cognitive Assessment; UPDRS‐III, part 3 of the Unified Parkinson's Disease Rating Scale (2003 version).

In Table 1, PDRP *z*‐scores, putamen DAT‐binding ratios, and TDI scores are shown for each RBD patient. This permits identification of several RBD subgroups. Subjects 1‐3 have normal values for all 3 markers. Subjects 17‐21 have abnormal values for all 3 markers: suprathreshold PDRP *z* scores, putamen DAT‐binding too low for age, and TDI scores < 18. Subjects 15 and 16 have suprathreshold PDRP *z*‐scores and abnormal DAT scans, but TDI scores ≥ 18. Of the 9 subjects with abnormal DAT scans, 7 had suprathreshold PDRP *z*‐scores (subjects 15‐21). Interestingly, of the 12 subjects with normal DAT scans, 5 (42%) had suprathreshold PDRP *z*‐scores (subjects 10‐14).

On average, subjects with abnormal DAT scans (n = 9) had higher PDRP *z*‐scores compared with subjects with normal DAT scans (*P* = 0.044, uncorrected). Subjects with olfaction scores < 18 (n = 9) had higher PDRP *z*‐scores compared with subjects with olfaction scores ≥ 18 (*P* = 0.032, uncorrected). Putamen DAT‐binding ratios were not significantly different between the 2 olfaction groups (*P* = 0.117). PDRP *z*‐scores, DAT binding, and olfaction were not significantly correlated, but trends were observed (n = 21; Supplementary Fig. 1).

## Discussion

Our findings underscore the value of the PDRP as a potential disease biomarker in idiopathic RBD. In line with 2 previous studies, RBD subjects significantly expressed the PDRP.^12^, ^13^ Although on average, PDRP *z*‐scores were lower in RBD subjects compared with PD/DLB, more than half of the RBD subjects already had a PDRP *z*‐score in the range of PD patients.

This study is the first to directly compare PDRP expression, striatal DAT binding, and olfaction in RBD. Although a trend was observed, PDRP and striatal DAT binding were not significantly correlated. Previous studies in PD have shown that PDRP expression shows only modest correlation to DAT binding.^10^, ^28^, ^29^ This may indicate a partly nondopaminergic genesis of the PDRP. Remarkably, 5 of 12 RBD patients with normal striatal DAT binding had suprathreshold PDRP *z*‐scores. In 2 of these cases, ^18^F‐FDG‐PET was performed before DAT‐SPECT. It has been shown that some DLB patients may initially have unremarkable DAT scans.^30^ It is possible that RBD subjects with significant PDRP expression but normal DAT binding will eventually develop DLB. Longitudinal imaging studies of RBD subjects are needed to further investigate the relationship between PDRP expression and loss of DAT binding in relation to the final clinical diagnosis.

That there was no direct significant correlation between PDRP *z*‐scores, DAT binding, and olfaction could indicate that the 3 markers provide complementary information. For example, 2 cases had suprathreshold PDRP *z* scores and abnormal DAT scans, but TDI scores ≥ 18. These subjects would have been considered at low risk of phenoconversion if the olfaction scores alone had been considered.^16^ We also identified 3 subjects with normal values for all 3 markers. These individuals may have a low risk of converting to PD/DLB. In contrast, 5 subjects had suprathreshold PDRP *z* scores, putamen DAT binding too low for age, and TDI scores < 18; these subjects may be considered to have a particularly high risk of conversion within the next 5 years.

The data presented in this report are cross‐sectional. A longitudinal study of our RBD cohort is ongoing. Follow‐up data will be essential to elucidate if DAT‐SPECT‐negative DLB cases, and perhaps subjects who later developed multiple system atrophy, contributed to the aforementioned findings. We expect that the PDRP will be especially informative, because in contrast to olfaction,^31^ the PDRP is a progression marker.^11^ Moreover, PDRP expression is useful in the differential diagnosis of parkinsonian disorders,^32^ whereas DAT imaging is not.^33^

## Author Roles

1) Research project: A. Conception, B. Organization, C. Execution; 2) Statistical Analysis: A. Design, B. Execution, C. Review and Critique; 3) Manuscript: A. Writing of the first draft, B. Review and Critique.

S.K.M.: 1ABC, 2ABC, 3AB

D.V.: 1BC, 3B

R.J.R.: 2ABC, 3B

E.S.W.: 1ABC, 3B

G.M.: 1ABC, 3B

C.D.: 1ABC, 3B

K.R.: 1ABC, 3B

S.O.: 1ABC, 3B

A.P.: 1ABC, 3B

F.E.R.: 1C, 3B

T. van L.: 1C, 3B

L.H.: 1ABC

L.K.T.: 1A, 3B

H.H.: 1ABC, 3B

M.L.: 1ABC, 3B

K.K.: 1ABC, 3B

S.M.A.: 2ABC 3B

J.B.: 2ABC, 3B

K.L.L.: 1ABC, 2ABC, 3B

W.H.O.: 1ABC, 2ABC, 3B

## FINANCIAL DISCLOSURE OF ALL AUTHORS

This study was funded by the Dutch Stichting Parkinson Fonds and the German Parkinson Fonds Deutschland.

S.K.M.: Stichting Parkinson Fonds (grant); D.V.: none; R.J.R.: none; E.S.W.: Parkinson Fonds Deutschland (grant); G.M.: none; C.D.: none; K.R.: German Federal Ministry of Education and Research (BMBF 01GQ1402; grant); S.O.: the Netherlands Organization for Scientific Research (grant 016.116.371), UCB Pharma, Boehringer Ingelheim, and Novartis (other); A.P.: UCB Pharma (other); F.E.R.: none; T.v.L.: Brittania (advisory boards and honoraria), neuroderm (advisory board), Medtronic, AbbVie (honoraria), Stichting Parkinson Fonds, Mosadex Pharma, EGRET European Fund, Weston Brain Institute, BCN Institute at University of Groningen (grants); L.H.: none; L.K.T.: none; H.H.: none; M.L.: Michael J. Fox Foundation for Parkinson's Research (grant).; K.K: none; S.A.M.: none; J.B.: GE Healthcare (grant); K.L.L.: Stichting Parkinson Fonds (grant); W.H.O.: BiogenIdec, Medigene, Merck Darmstadt, Roche (stock ownership in medically related fields), Adamas, BristolMyerSquibb, GE Health, Mundipharma, Novartis, Roche, Teva, UCB (advisory boards), AbbVie, Desitin, Lundbeck, Mundipharma, Novartis, UCB (honoraria), German Ministry of Education and Research, German Research Foundation, Michael J. Fox Foundation, Internationaal Parkinson Fonds, Novartis Pharma Germany (grant).

## Supporting information

Additional Supporting information may be found in the online version of this article at the publisher's website.

Supplementary Information
