# Supplementary material for: FDG PET, dopamine transporter SPECT, and olfaction: Combining biomarkers in REM sleep behavior disorder
Source: Mov Disord. 2017 Jul 22;32(10):1482–6. doi: 10.1002/mds.27094 (PMC5655750; doi:10.1002/mds.27094)
Supplement: Supplementary file 1 — Supplementary Information [file MDS-32-1482-s001.docx]

Supplementary Table: Group Averages of Clinical Information and Comparison to Controls

|  | Controls (n=19) | RBD (n=21) | P-value^*^ |
| --- | --- | --- | --- |
| Age (years) | 62.4±7.5 | 61.9±5.4 | 0.82 |
| Gender (male/female) | 9/10 | 18/3 | 0.010 |
| Age at onset of RBD |  | 55.0±7.1 |  |
| RBD duration (years) |  | 6.9±5.4 |  |
|  |  |  |  |
| MoCA | 28.3±1.6 | 26.4±1.9 | 0.003 |
| UPDRS-III | 0.8±1.25 | 2.6±2.0 | 0.002 |
| Olfaction (TDI score) ^†^ | 33.3±5.1 | 19.0±11.3 | <0.001 |

Values are mean ± standard deviation unless otherwise specified.

^*^Independent T-test for age and PDRP z-scores, Chi^2^ test for gender, Mann-Whitney U test for MoCA, UPDRS-III, and olfaction. Uncorrected P-values are shown.

^†^Olfaction was measured with Sniffin’ Sticks. In this test, the olfactory threshold (T), discrimination (D) and identification (I) of smells is tested. Total scores summing these three aspects (TDI) are reported.

Supplementary Figure 1. Correlations between PDRP, DAT-binding, and Olfaction in RBD subjects (n=21)


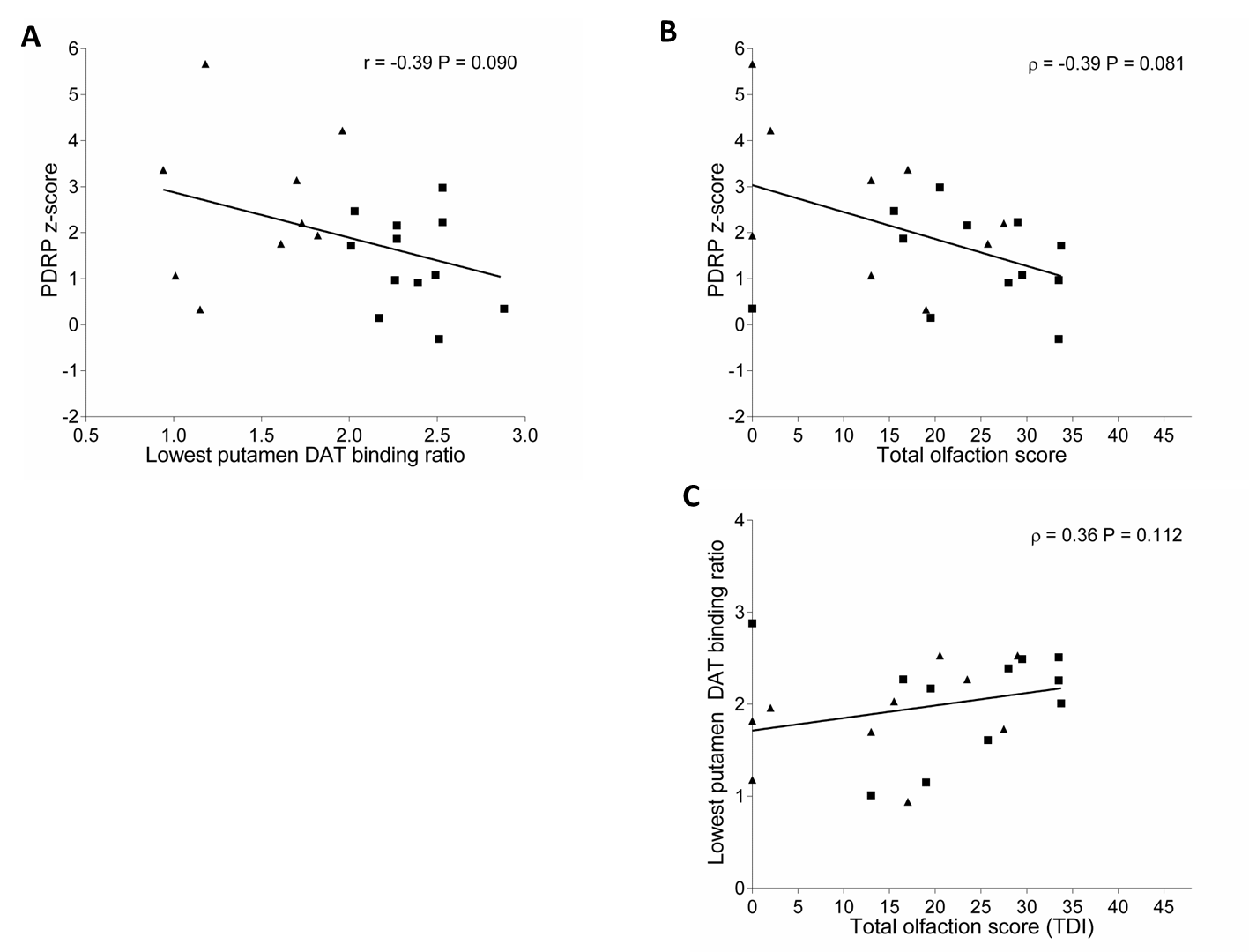


No significantly correlation was found between PDRP z-scores and putamen DAT-binding ratios (although a trend was observed – see graph A). Neither PDRP z-scores and olfaction (graph B), nor DAT-binding and olfaction (graph C) were significantly correlated. In graphs A and B, triangles indicate abnormal DAT-binding. In graph C, triangles indicate supra-threshold PDRP z-scores.

Supplemental Material: Detailed Methods of DAT-SPECT

RBD subjects (n=21) underwent DAT imaging with ¹²³I-2β-carbomethoxy-3β-(4-iodophenyl)-N-(3-fluoropropyl)nortropane (¹²³I-FP-CIT) SPECT in Marburg, Germany as described previously (15). ^123^I-FP-CIT binding in striatal regions was quantified with The Brain Registration & Analysis Software Suite (BRASS™, HERMES Medical, Sweden). Specific to non-specific binding ratios were calculated in the caudate nucleus and putamen bilaterally, using the occipital cortex as reference (i.e. non-specific binding).

To determine whether binding ratios were abnormal for age, 24 healthy controls were used for reference (age range 18-74 years). Because these controls were scanned on a different camera (NeuroFocus system (software upgrade of the Strichman Medical Equipment system; Massachusetts, USA)), phantom measurements were performed to calculate a correction factor for our system (Siemens Symbia S, Low Energy High Resolution) to match the reference dataset. A striatal phantom (RS-901T; GE) was used for direct, quantitative comparison between the two SPECT systems. Binding ratios which were 2 or more standard deviations lower than age-matched expected control values were considered abnormal. Before analysis, all scans were anonymized to the reader.
